# *Plasmodium falciparum* in Asymptomatic Immigrants from Sub-Saharan Africa, Spain

**DOI:** 10.3201/eid1802.111283

**Published:** 2012-02

**Authors:** Begoña Monge-Maillo, Francesca Norman, José Antonio Pérez-Molina, Marta Díaz-Menéndez, Jose Miguel Rubio, Rogelio López-Vélez

**Affiliations:** Ramón y Cajal Hospital. Madrid, Spain (B. Monge-Maillo, F. Norman, J.A. Pérez-Molina, M. Díaz-Menéndez, R. López-Vélez);; National Centre of Microbiology, Madrid (J.M. Rubio)

**Keywords:** malaria, immigrants, asymptomatic infections, parasites, public health, Spain, Africa, sub-Saharan Africa, screening

**To the Editor**: A range of infectious diseases have been described in asymptomatic immigrants (*1*), which may justify the implementation of screening after obtaining consent. In particular, asymptomatic malaria caused by *Plasmodium falciparum* parasitemia among recently arrived immigrants may be a major public health problem outside malaria-endemic areas because these patients may be involved in autochthonous transmission cycles and may act as reservoirs capable of reintroducing malaria into areas where it had been previously eradicated.

In 2010, we reviewed the medical records of 314 asymptomatic (defined as patients with no symptoms at the time of consultation) immigrants from sub-Saharan Africa who had settled in Spain, had not traveled to their countries of origin since arrival, and had been examined at the Tropical Medicine Unit (TMU) of the Ramon y Cajal Hospital in Madrid during the previous 5 years. Systematic screening included a blood count; serum biochemistry; basic urine analysis; serologic tests for HIV infection, hepatitis B or C infection, syphilis, and schistosomiasis (if epidemiologic risk factors were present); tuberculin skin test; analysis of fecal samples for parasites; and PCR to identify *Plasmodium* spp (*2*). Date of arrival in Spain was obtained from the patient and corroborated by the nongovernmental organizations caring for them.

PCR for *Plasmodium* spp. was performed for 216 patients, and 10 (4.6%) had positive test results for *P. falciparum*. Nine were men; median age was 27 years (interquartile range [IQR] 20–31 years). The median period from arrival in Spain to malaria diagnosis was 4.5 months (IQR 1.75–12.5 months). Three men received a diagnosis of *P. falciparum* malaria >1 year after arrival.

 Patient 1 was a 32-year-old man from Angola who came to the TMU for screening 13 months after arriving in Spain. He was treated with a standard regimen of artesunate plus sulfadoxine/pyrimethamine; latent tuberculosis (TB) infection and schistosomiasis were also diagnosed. Patient 2 was a 17-year-old man from Senegal, seen at the TMU 14 months after arrival. Malaria treatment was not prescribed because he was lost to follow-up. He was also diagnosed with latent TB infection. Patient 3 was a 28-year-old man from Guinea who visited the TMU 28 months after arrival in Spain. He was treated with a standard regimen of atovaquone/proguanil and also received diagnoses of tuberculosis (TB) infection, schistosomiasis, and strongyloidiasis. No statistically significant association was observed between positive or negative PCR for *P. falciparum* and a diagnosis of tuberculosis (TB) infection, hepatitis B or C virus infection, HIV infection, syphilis, intestinal parasite infection, or schistosomiasis*.* None of the 3 patients had received a blood transfusion since arriving in Spain.

Reported prevalence of imported malaria among immigrants may be >10%, according to some studies (*1*), with higher rates among persons from sub-Saharan Africa (malaria caused by *P. falciparum* occurring mainly 3 months after arrival). Clinical symptoms of malaria in immigrants are typically mild, with low levels of parasitemia. Many immigrants may be asymptomatic (*1*,*3*), which has been explained by partial immunity acquired gradually after prolonged exposure in areas with stable malaria transmission. Because infected persons may initially have no symptoms, implementation of malaria screening for recently arrived immigrants from disease-endemic areas would seem advisable (*4*).

How long a low level of *P. falciparum* parasitemia may persist once exposure to malaria has been discontinued is not known. Mathematical models have estimated the maximum duration of *P. falciparum* infection after interruption of transmission at ≈4 years (*5*), although delayed clinical presentations of *P. falciparum* malaria have been described as long as 2 (*6*), 4 (*7*), or even 8 years (*8*) after patients have left malaria-endemic areas. These data highlight that low asymptomatic parasitemia may persist long after migration.

Determining in which patients with asymptomatic parasitemia clinical malaria will develop and when, or if, any external factors may act as triggers would also be useful. A study in France found that 2.3% of malaria cases among immigrants developed >1 year after their arrival and that pregnancy and co-infection with HIV were factors associated with late presentation of malaria caused by *P. falciparum* (*9*).

Asymptomatic malaria cases may affect public health in non–disease-endemic areas because persons with low-grade parasitemia are capable of infecting mosquitoes (*10*). These persons could act as unidentified reservoirs and contribute to transmission in areas where malaria has been eliminated*.* In addition, congenital transmission or transmission by blood transfusion or organ transplantation may occur even when the donor has lived for years outside the malaria-endemic area.

Our cases highlight how malaria parasites may persist in asymptomatic immigrants long after their arrival in the host country (up to 28 months). On the basis of published reports of symptomatic delayed cases, we believe that the prevalence of persistently low-level parasitemia among asymptomatic immigrants is probably higher than previous estimates. Screening for malaria among immigrants long after arrival would help determine if there are any factors that influence the development of clinical malaria. Delayed screening could also be particularly relevant in certain risk groups, such as pregnant women and persons who are HIV positive. As a public health measure, such delayed screening could play a role in preventing outbreaks or reintroducing malaria in countries where it has been eradicated.
